# Editorial: introducing dedication reviews—broad reviews in plant and horticultural sciences

**DOI:** 10.1093/hr/uhae358

**Published:** 2025-01-06

**Authors:** Zong-Ming (Max) Cheng

**Affiliations:** College of Horticulture, Nanjing Agricultural University, Nanjing 210095, China

*Horticulture Research* is pleased to announce the launch of ‘Dedication Reviews’, a new article type aimed at publishing comprehensive reviews that summarize the history of a specific area of research and highlight recent developments or discoveries within the plant sciences and horticulture, with special dedications to key individuals. We envision two general types of reviews in this category.

The first type will cover topics across the broad spectrum of plant science, designed to appeal both to active researchers in plant sciences and to our diverse readership in horticulture. These reviews are typically authored by scholars focused on fundamental plant biology, such as growth and development, environmental interactions, genomics, evolution, domestication, bio- and abiotic stress responses, and mechanisms underlying global environmental change, secondary metabolism, etc. While the discoveries in these areas often stem from research on model plants, agronomic crops, or ecological (non-domesticated) species, we encourage authors to include horticultural crops wherever applicable. In cases where this is not possible, authors should explicitly connect their review to horticulture by discussing how the reviewed topic can inform horticultural practices, with emphasis on its potential applications and impact. This relationship is crucial, as horticultural crops represent a highly diverse yet specialized group of domesticated plants that play a vital role in human food, nutrition, environmental aesthetics, and plant-based medicine. Furthermore, horticultural crops span from lower to higher plants, and nearly all plants have potential uses in horticulture, whether as fruits, vegetables, ornamental species, or dual-purpose plants for medicinal applications.

The second type of review will likely be led by horticultural scientists specializing in a single or small group of commodity crops, such as stone fruits (e.g. *Prunus*), pome fruits (apple and pear), citrus, melons, tomato, cabbage, roses, and others. These reviews will focus on the progress and recent discoveries related to these crops, aiming to developing optimal varieties for human or industrial use. In such reviews, a brief introduction to the crop’s industry and its significance should be provided to engage non-specialist readers. The review should then explore advances and challenges in various scientific areas, including genetics/genomics, growth and development, produce quality, and biotic and abiotic interactions. We also encourage authors to reflect on how research focused on a single or a group of horticultural crops can impact broader plant science and be applied to other species.

These two types of ‘Dedication Reviews’ are intended to foster interaction between plant biologists and horticultural scientists, encouraging a deeper understanding of each other’s research interests, concerns, and methodologies. Through this dialogue, specialized horticulturists can learn how advances in broader plant science can be applied to horticulture, while plant scientists may be inspired to collaborate with horticulturists or shift their focus to crops of horticultural significance. This vision was outlined in my inaugural editorial when *Horticulture Research* was launched in 2014 [1]. To support this goal, we encourage authors to present a wide range of opinions from the literature, critically evaluate especially underrepresented studies and controvertible opinions, and provide thought-provoking discussions and perspectives. Merely summarizing or listing studies without critical analysis is discouraged. We aim for these reviews to unite leading experts in the field to produce authoritative works that will serve as a starting point for future research.

As *Horticulture Research* is an open-access journal, the reviewed content, including figures and tables, will be freely available. We hope that these high-quality reviews will be widely adopted for educational purposes, from undergraduate and graduate programs to high-school biology teaching.

In line with the title ‘Dedication Reviews’, each article will feature a section titled ‘Dedication’ (typically no longer than one page), in which the author(s) explain who the review is dedicated to and why. We envision two types of dedications: the first is to a prominent scholar who has made a significant contribution to the field of the review; this is generally made by senior, established authors. The second type is dedicated to individuals who have had a significant impact on the author’s career, typically junior authors. In cases where both senior and junior authors collaborate, both types of dedications may be included. The purpose of this section is twofold: to recognize pioneering scholars whose work has advanced the field but may not be widely known to newcomers, and to acknowledge mentors who have played a crucial role in shaping the authors’ careers. This practice aims to foster a culture of mentorship and recognize the often-overlooked contributions of mentors in scientific education and training.

I am very grateful that Dr. Harry Klee, Professor Emeritus of University of Florida (hjklee@ufl.edu), has agreed to lead ‘Dedication Reviews’. Dr. Klee and I will assemble an outstanding editorial board, who, along with the Associate Editors, advisory board members, and Emeritus Editors of *Horticulture Research*, will actively commission these reviews from scientists engaged in cutting-edge research, both from the perspective of plant biology and horticultural commodities. Proposals or inquiries about reviews are always welcome. Authors are encouraged to consult similar reviews published in *Horticulture Research* within the last 2–3 years, as repetitive topics will be discouraged. Once a proposal is approved, the author(s) will be invited to submit a full review, which will undergo a rigorous peer-review process. Only the highest quality reviews will be accepted for publication.
